# Book Reviews

**Published:** 1824-12

**Authors:** 


### CRITICAL ANALYSIS

OF

### ENGLISH AND FOREIGN LITERATURE

RELATIVE TO THE VARIOUS BRANCHES OF

### Medical Science.

Quæ laudanda forent, et quæ culpanda, vicissim

Illa, prius, cretâ; mox hæc, carbone, *notamus*.—PERSIUS.

### DIVISION I.

### ENGLISH.

ART. I.—*Commentaries on Diseases of the Stomach and Bowels of Children.* By ROBLEY DUNGLISON, M.D. Lecturer on Midwifery and the Diseases of Women and Children; Member of the Royal Academy of Marseilles; of the Royal Society of Sciences, Arts, Belles Lettres, and Agriculture, of Nancy; of the Society of the Faculty of Physicians; the Pharmaceutical and Linnæan Societies of Paris; the Physico-Medical Society of Erlangen; the Academic Medical Society of Marseilles; Secretary for Foreign Correspondence to the Medical Society, and Member of the Hunterian Society of London; Consulting Accoucheur to the Eastern Dispensary, &c. &c. —8vo. pp. 201. Whittaker, London, 1824.

IT must be evident that, in the investigation of the diseases of infancy, there are great and peculiar difficulties to surmount. From the little patient no information can be gained, and so completely is the frail and tender body the sport of a thousand apparently unimportant circumstances, that it requires no ordinary professional acuteness to keep the eye fixed upon the grand features of disease, and not to be misled by symptoms which are only occasional and evanescent. We must not, however, approach the treatment of the diseases incidental to a very early age, with the depressing and erroneous conviction, that from practice and industry no benefit is to be derived; although we ought to be duly impressed with the difficulties to be contended with, that we may be induced to bring into action all our attention and consideration to overcome them. "To the experienced eye," says our author, "the deviations from health are readily recognised. The state of the skin, secretions, excretions, tongue, eye, breath, fatness, or leanness, gestures, cries, &c. &c. will sufficiently indicate the difference between the healthy and a diseased condition."

We readily admit the practical tact, that the frequent and attentive observation of diseases will impart; and we are indebted to M. JADELOT, physician to the Hôpital des Enfans trouvés, for the communication of his experience. In the prefatory observations of our author, the opinions of M. Jadelot, which he has embodied under the term of "La Séméiologie physiognomonique," are briefly stated. They have been published in a work of M. DE SALLE, who estimates the proposed mode of discriminating the diseases of childhood so highly, that "he conceives their diseases will be recognised with such facility, that the possessor of the most mediocre talent may know and cure them as readily as he who is more highly gifted." It is, of course, yet to be determined whether the physiognomic investigation of a sick child will ever place the "most mediocre talent" and the more "highly gifted" upon a practical level. We hope it may, for we wish well to the multitude; but we fear it never will.

The following is a brief detail of the doctrine professed by M. Jadelot.—"Three principal traits are observable on the countenances of young children. These are nearly parallel, and uniformly proceed from the middle towards the lateral and inferior part of the face. The first commences at the greater angle of the eye, and is lost a little below the projection formed by the cheek-bone: this is called the *oculo-zygomatic.* The second begins at the upper part of the ala nasi, and embraces, in a semi-circle more or less perfect, the outer line of the orbicularis oris. It is not uncommon to observe, towards the middle of the cheek, and forming a species of tangent with the trait just described, another, which in certain faces constitutes the dimple of the cheeks. The alterations of these two traits are referable to similar affections: the one he terms *nasal,* the other *genal.* The last begins at the angle of the lips, and is lost on the lower portion of the face: this is called the *labial.* It seldom forms a deep line, being modified by the changes which the neighbouring parts undergo: the others, on the contrary, are more or less deeply marked, according as the diseases to which they are referable are more or less intense or chronic. The first-mentioned trait is the index of disorders of the cerebro-nervous system; the second, and its accessory, signalise those of the digestive passages, and of the abdominal viscera; the third is the concomitant of diseases of the heart and air-passages. Speaking generally, they are all the outward signs of lesions of the great splanchnic cavities. The oculo-zygomatic trait is strongly marked in all those diseases whose primary seat is in the brain and nerves. It is likewise observable whenever these organs actively participate in affections which were, in the first instance, foreign to them; but at such times a coincidence of some other facial line is found, which indicates the complication. Thus, for instance, it is said that the moment at which inflammation of the lungs, or catarrh, becomes converted into neurosis, or at which a common cough becomes that of pertussis, is marked upon the child's countenance by the oculo-zygomatic trait. The same circumstance occurs when any disorder, primarily seated in the intestines, affects the brain; as, for example, when the presence of worms, or of chronic inflammation in the digestive tube, occasions epilepsy, convulsions, or hydrocephalus."

At the onset of all severe diseases, according to M. de Salle, the inspection of the child's countenance may serve as a useful guide to the physician in discovering the organ affected. Of the truth of this opinion there can be no doubt; and it is not in children alone that information may be thus obtained by an attentive observer, for in adults also it is of the utmost importance in assisting our diagnosis. No man can have watched a patient labouring under inflammatory affections of the abdomen, without being struck by the forcible and peculiar character impressed upon the countenance: "*le cri des organes qui souffrent*," as Broussais has fancifully styled it. The subject is worthy of the most deliberate investigation, and we think promises a rich harvest of practical information; but we forbear from further observation upon it on the present occasion, as a work of M. Jadelot's, on "Séméiologie Physiognomonique," is on the eve of appearing, which we shall not allow to escape without due attention. We perfectly agree with our author, that plates should be given to elucidate his different positions. To fix upon the mind, by verbal description, an impressive sketch of various changes of features, is as impossible as it is to convey a clear notion of cutaneous diseases without graphic illustrations.

We now enter upon the first chapter of the "Commentaries" of Dr. Dunglison, containing the consideration of *Intestinal Worms.* "At one period of medical history, there was scarcely a disease, the origin of which was not referred to the influence of animalculæ in some form; and even at the present day, on the continent, invermination is considered to have a much more extensive agency in the production of disease, than is allowed to it on this side the Channel, and more than it appears to be fairly entitled to." There is one species of animal, however, which has of late years been thought, by some among us, to have an important influence in the production of several diseases,—viz. the hydatid. "To this insect," says our author, "pulmonary and other tubercles, the various tumors affecting the corporeal fabric, have been ascribed; but no clear evidence has, as yet, been adduced to render the notion indisputable." The work of Dr. Baron, on Tuberculated Accretions, is not mentioned by our author, although that gentleman supports his opinion of the frequent conversion of hydatids into tubercular masses by much solid reasoning, and numerous interesting facts.

Dr. Dunglison considers the best systems for the classification of the worms which infest the human body, to be those of RUDOLPHI and BREMSER, from whose united labours FRANK has taken his arrangement. A detailed description of the characteristic structure of each species of worm is given. "With respect to the mode in which worms are generated in the human body, various opinions have been entertained. The chief controversialists may, however, be divided into two great classes,—viz. those who believe in equivocal generation, and those who consider that the germs must be always received from without." The symptoms which indicate the presence of worms in the human body, are very obscure. "It has, indeed, by some been affirmed that they are perfectly innocuous; whilst others have, on the contrary, ascribed almost all the diseases to which mankind is subjected to this agency. Both these sentiments are erroneous: there cannot be a doubt but that the health is occasionally very much injured by them; whilst they are not entitled to such extensive agency in the causation of disease, as has been by many imagined. Some years ago, Frank was requested to see a prince who had been attacked with epilepsy: his physician assured him that he could make him void, at pleasure, thousands of filiform worms; but, as he was neither able to define the genus nor species of these worms, Frank requested to witness the phenomenon. The physician administered a dose of castor-oil, which produced many stools, in which were thousands of whitish filaments, similar to small eels; but, on an attentive examination of these pretended worms, they were found to consist entirely of the castor-oil in a state of coagulation."

The general symptoms which denote, or are believed to denote, the presence of worms, are well known; yet it may not be useless to give a rapid sketch of the phenomena, described by the continental writers as occurring in an exquisite case of this kind, for which M. ALIBERT has proposed the appropriate name of *helminthiasie*. The face is swelled, pale, and sometimes livid; the integuments under the lower eye-lid become of a leaden hue; there is itching or tension of the nose, occasionally with epistaxis; during sleep, the saliva runs from the mouth; the breath is offensive; there is grinding of the teeth, particularly at night; aphonica. Squinting or other distortion of the eyes, with dilated or even immobile pupil; sometimes with amaurosis. Unpleasant dreams; moroseness and obstinacy of temper; lastly, chorea, risus sardonicus, vertigo, delirium, and stupor. All these symptoms are more or less referable to the head. With regard to the chest, we have frequent dry cough; anxiety at the præcordia; occasional stitches, resembling pleurisy; hiccough, and other convulsive affections of the diaphragm. The sensation of something creeping from the stomach along the œsophagus, is occasionally felt; and, more rarely, worms presenting themselves at the nose or mouth. Frank has even known them produce suffocation, by falling into the larynx.

In the abdomen, we find, with regard to the stomach, that the appetite is sometimes insatiable, although it is attended by progressive and rapid emaciation; while at other times nausea, retching, and cardialgia, prevail. With respect to the bowels, a sense of cold, gnawing, or tearing; borborigmi; tympanitic swelling; eructations; tormina; tenesmus, and discharge of mucus from the rectum, and occasionally of the debris of rotten worms.

The question whether worms ever perforate the intestines, is one of some interest; and Frank states, in reply, that, in the course of fifty-four years' practice, he only met with one such example, out of several thousand bodies which he examined. The cases of this kind, related by various writers, are therefore probably to be attributed to accidental ulceration of the parts, through which the worms have made their escape; an opinion in which both Rudolphi and Bremser concur.

Upon the whole, we are compelled to acknowledge, that, after all the labour which has been expended to determine the symptoms which indicate the presence of worms, the only true pathognomic sign is their expulsion. We possess no satisfactory information of the symptoms which characterise the presence of any particular species.

Frank has paid great attention to verminous affections; and Dr. Dunglison gives the following English version of his statement:

"In enumerating the symptoms of verminous affections, I have pointed out their prognosis. It has been shown that the symptoms produced by the presence of worms, are at one time trivial, at another severe; and that, in some cases, from their occasioning no inconvenience, there is no evidence of their existence. Intestinal worms are more exposed to the action of remedies, adapted for their destruction, than those which fix their residence in the parenchyma of other viscera. They find themselves, however, protected by the mucus which lines the alimentary canal, by the fecal matters with which they are enveloped, and by the folds of the mucous membrane in which they conceal themselves. At other times, as is frequently observed in cases of tænia, the head is firmly implanted in the tissue of the intestines. It is consequently not astonishing that, in a considerable number of cases, they are but little sensible to the action of anthelmintics, and obstinately resist these remedies; or that the articulated worms should suffer only the loss of some of their rings, which is speedily repaired. Occasionally, they seem to sport with the physician: after having braved his efforts, they make their exit spontaneously; but commonly leave, in the intestines, germs which perpetuate their species, and compensate for their voluntary disappearance.

"All the species of intestinal worms do not offer the same resistance to the means employed by the physician: in general, the *ascarides lumbricoides* yield readily; the *oxyures vermiculares* are more difficult of expulsion; the *tænia solium* is still more obstinate; whilst the *bothriocephalus latus (tænia lata)* is almost invincible, and requires a treatment which sometimes endangers the life of the individual affected with it. Induced by these observations, or led away by a spirit of novelty, some authors have confirmed the old proverb, '*nihil est tam absurdum quod non docuerit philosophus,*' by maintaining that worms are intended for absorbing the superfluous mucus of the intestines, to increase their peristaltic action, by the irritation which their presence occasions in the structure of these organs; and, consequently, that they are useful to man, instead of being injurious to his health. It must be confessed that the predisposent causes of worms exercise the greatest influence in the production of diseases which are attributed to those animals; that an infinite number of persons carry worms in their intestines during the whole course of a long life, without being much incommoded by them; that physicians sometimes gratuitously conceive, to the prejudice of the patient, the existence of these animalculæ; and that, in several cases, (more commonly, indeed, than is imagined,) where they really do exist, they are not the cause of the disease which is ascribed to them. The symptoms, however, induced by worms themselves, prove that the morbid affections attributed to them are not always chimerical. In children, women, and men of a delicate constitution, worn down by enervating causes, labouring under different severe diseases,—as scarlatina, variola, and measles,—worms frequently exert great ravages, and even occasion death. Nor are they less to be feared when they attack organs essential to life. The *ascarides lumbricoides,* which are so common, produce more unpleasant symptoms when they ascend into the stomach, than when they remain in the intestines. When worms creep out spontaneously by the mouth or anus, at the end of fevers, they are doubtless driven away by the morbid heat, or by the hunger which they feel, owing to the patient's taking no food. They are also observed to creep out at the approach of death, during the time of the agony, and when the patient has rendered his last sigh. Hippocrates regarded the exit of worms at the termination of a disease, as a favourable presage:—'Lumbricos teretes, morbo judicium subeunte, una cum excrementis prodire, utile erit." This occurrence, however, I have frequently witnessed, without either a favourable termination having followed, or any change having been induced in the progress of the disease." (P. 45, 48.)

The grand predisponent cause of worms, is a want of due tone and vigour in the system, and in the digestive organs more especially; and, in the treatment of verminous affections, therefore, the principal attention must be directed to the removal of these. "Without this important desideratum, we may destroy

2

by anthelmintics one set of these parasites, but others will continue to be produced. The subject of verminous disease should, therefore, respire a pure air, take proper exercise, avoid the use of crude, indigestible nourishment; and, in short, adopt every means of improving the tone of the system." A decisive vermifuge process is yet a desideratum in medical practice. "Worms in the intestinal canal are so involved in mucus, that remedies, which readily destroy them out of the body, are considerably mitigated in their action on those inhabiting the human frame; whilst some of those that are recommended have a tendency, at the same time, to weaken the tone of the stomach and bowels, and thus, by augmenting the predisponent cause, to increase the disease." Violent drastics are strongly deprecated, although a brisk cathartic is regarded as a valuable agent. Only two mechanical anthelmintics are exhibited at the present day, —filings of tin and the cowhage; and the testimonies in favour of both are numerous. Most of the true anthelmintics which have been recommended, are almost, if not wholly, inert. Oil of turpentine, simply or combined, and the *semina santonicæ*, or wormseed, are, in the opinion of Dr. Dunglison, the only two medicines of this class it is necessary to retain.

The following is the plan adopted by Bremser, being similar to that employed by Frank, in the early stages of the disease:—

"He commences with the following electuary:

℞. Seminum Cinæ (Santonicæ) aut Tanaceti vulgaris, ruditer contusorum, ℥ss.

Pulveris radicis Valerianæ sylvestris, ʒij.

——— Jalapæ, ʒss. vel ℈ij.

Potassæ Sulphatis, ʒiss. vel ʒij.

Oxymellis Scillæ, q. s. ut fiat Electuarium.

The patient is recommended to take two tea-spoonfuls of this remedy twice or thrice a-day, until the whole is consumed.

"When this is finished, he gives, morning and evening, two dessert-spoonfuls of the empyreumatic oil, and directs the mouth to be rinsed afterwards with a little water. Should the oil act too powerfully on the nervous system, or on the bladder, the dose must be diminished. According to this plan, about two ounces and a half will be taken in the space of ten or twelve days. The following purgative is then exhibited:

℞. Pulver. radicis Jalapæ, ℈j.

Folior. Sennæ, ʒss.

Potassæ Sulphatis, ʒj. Misce.

This powder is directed to be taken every hour, until full evacuations are produced; after which the oil must be again resumed, and persisted in until four, five, six, and even eight, ounces shall have been taken, according to the difficulty [illegible]ich may be experienced in expelling the tænia, or other entozoa." ([illegible]. 74, 75.)

Among the external applications used for anthelmintic purposes, the only one remarkable for its novelty,—we can scarcely say, for its elegance or practical utility,—is assafœtida dissolved in gastric juice! This wise recommendation comes from M. Cloquet: how a supply of gastric juice is to be obtained, he has not thought proper to inform us.

Let it, however, always be borne in mind, that we depend upon a tonic mode of treatment for the annihilation of the predisposition to worms.

We do not feel ourselves called upon to occupy the attention of our readers, by a lengthened detail of the contents of the remaining chapters of the volume. To us they appear somewhat uninteresting, although we are not prepared to deny the accuracy of many facts which are gravely stated: for example, —that "the retention of the meconium will produce disturbance in the infant; that a few grains of rhubarb, or a teaspoonful of castor-oil, may be given to act upon the bowels, if necessary; and that nurses sometimes apply a piece of butter, mixed with soft sugar." These are truths, which may not altogether have escaped the notice of our readers.

In the chapter on *Constipation*, we find that, according to Tissot, spasm of the sphincter ani muscle is not unfrequently the cause of retention of the meconium; and the hint is, at least, worth attending to. In such cases, he regards purgatives as injurious, and advises the use of emollient fomentations, the warm bath, &c. When the constipation is rather obstinate, our author recommends the introduction of a suppository into the rectum, formed of yellow soap: this he thinks equally efficacious, and more manageable than clysters. Upon the whole, we regard Dr. Dunglison as too partial to purgatives of the drastic kind, particularly scammony, calomel, and aloes: of the latter he says—

"In some very obstinate cases, I have found the administration of aloes in powder succeed in emptying the intestines, where all the common remedies had been ineffectually employed: it is, indeed, in such cases only that its exhibition, to the extent which I have used it, can be justified.

"I was first induced to employ this medicine so largely, from the very high eulogums I had heard pronounced upon it by Dr. Hamilton, the present celebrated Professor of Midwifery in the University of Edinburgh, to whom the idea of administering it was suggested by observing, in a laboratory where he had been placed by his father, for the purpose of being instructed in pharmacy, that the syrup of buckthorn (so called), which they were in the habit of vending to mothers of families to be given to their children, was usually formed extemporaneously of aloes dissolved in treacle; and, upon making inquiries of those who had purchased it, he found that no bad effects had resulted from its administration. He consequently formed the determination of trying it in his own practice; when he found it to be not only a successful agent, when other means had failed, but also that it was rarely rejected by the stomach, acted mildly, was perfectly safe, and but seldom objected to by young infants. To older children, however, in whom the taste generally becomes exquisitely sensible, the last observation does not generally apply. In all the encomiums passed upon the use of aloes as a purgative, I can cordially concur. In some cases of constipation, and in others of diarrhœa, apparently occasioned by the retention of feculent matter in the upper portion of the intestines, I have seen its administration productive of the most happy effects. It has been but rarely objected to by children; and its use has never, to my knowledge, been attended with griping or any other unpleasant symptom." (P. 85—87.)

We confess, however, that we are much disposed to give the preference to the milder cathartics in young children; such, for example, as the following prescription, which Jadelot asserts "*ne manque jamais son effet.*"

℞. Fol. Sennæ, ʒiij.

Sodæ Sulphatis, ʒij.

Mannæ, ℥j.

Aquæ, ℥iv.

Sennam in aquâ bulliente per horæ quadrantem infunde, tum cola et adde Salem et Mannam."

A tea-spoonful of this is to be administered occasionally, until the bowels are opened.

The chapter on *Acidity, Flatulence, and Colic,* affords nothing to extract, either from the author's own opinions, or those of the numerous writers to whom he refers. That those evils proceed from errors of diet and irregularities of the bowels, and are to be remedied by change of the former and attention to the latter, can scarcely be regarded as information of absolute novelty; any more than the recommendation of M. Leroy, to half-roast a child's belly before a flaming fire, in order to cure it of the gripes, can be looked upon as an injunction of absolute wisdom. According to this practitioner, it must be "un feu qui fournit une belle flamme;" and, to make it more decided that he trusts not to the heat alone, he adds, "une chaleur portée au même degré, mais sans l'influence de la lumière, ne produit pas cet effet vivifiant."

The discussion on *Diarrhœa* is very lengthy, and the more interesting part relates to that form of disease consisting in softening, or complete ulceration, of portions of the intestinal canal, which constitutes the subject of the interesting paper by Mr. North, in the preceding part of this Number. Dr. Dunglison furnishes us with no new materials, and the chapter is almost entirely occupied by copious quotations from the work of M. Cruveilhier, and the papers of M. Andral, already referred to,—both of which have been analysed in former Numbers of our Journal. We cannot, however, refrain from expressing our astonishment that the author, compiling as he has done from so many foreign writers, should have entirely omitted, even to mention, the excellent paper of Dr. GAIRDNER, in the Medico-Chirurgical Transactions of Edinburgh, in which there is more information than in all the others put together. We would not have noticed this circumstance,—originating, we trust, in accidental omission,—had we not observed that some of our brethren have an extraordinary predilection for every thing that is foreign: as if French doctrines, like French wine, were more pure and digestible than those of English manufacture.

The opinions of Dr. Dunglison and various practical writers, with regard to a very formidable disease, are detailed in the following quotation:

"For that extremely dangerous variety of the disease to which the term *watery gripes* has been applied, it is difficult to lay down a plan of treatment adapted to all cases. Whilst many practitioners strongly inculcate the necessity of repeated purging, on a presumption of the cause being referable to the presence of irritating matter in some portion of the intestinal tube; others, equally exclusive, imagine it to be owing to an inflammatory condition of the mucous tunic, and to require the use of sedative remedies only. Were the causes properly appreciated, both classes of practitioners would probably be found occasionally right, and both occasionally wrong. I have sometimes witnessed cases in which there has been every reason for supposing that some accumulation had taken place in the cæcum, or small intestines; and where the increased peristaltic action, which had been the consequence, had produced irritation of the mucous coat, and augmented secretion of fluid by the exhalants. In such instances, of course, the affection could not be annihilated until after the removal of the offending matter. In other cases, purgatives have appeared to afford no relief, and the disease has rapidly yielded to the use of anodynes and the testacea. Opiates should, however, be administered with the greatest caution. I have myself lately witnessed two cases, in one of which a drachm of the syrup of poppies, and in another a powder containing a quarter of a grain of opium, proved fatal to young infants; and a case is referred to by the late Dr. Clarke, in which forty drops of Dalby's Carminative was attended with equally disastrous results.

"The opinions of those who have considered the disease to originate in retained fæces, have seemed to be confirmed by the circumstance of no solid matter being perceptible in the motions after its setting-in. This is, however, no proof whatever of the correctness of their discrimination. In common cases of diarrhœa, whilst the secretion of fluid is moderate, solid feculent matter may be perceptible; but, where the stools are frequent and the secretions immoderate, although in the aggregate the quantity evacuated may be equally great, it becomes so broken down and divided, as to present but little traces of it; so that its apparent paucity in any single evacuation, under such circumstances, ought rather, perhaps, to be considered as a gauge of the extent of the disease, than as a proof of retention. If this idea be correct, and it has appeared to me to have been borne out by observation, the practitioner might go on exhibiting purgatives, under the delusive hope of inducing a discharge of retained fæces, until he occasioned a degree of inflammation of the mucous membrane, or an increase of the colliquative discharge, from the effects of which the patient might not subsequently recover. Many cases, I have but little hesitation in asserting, have owed their fatal termination to this practice. But it may be replied, that, under such treatment, the feculent matter has re-appeared, and the patient's health immediately begun to be restored. Such reappearance, however, is no positive evidence of fecal retention having existed in those cases where the stools are extremely frequent, and dependent upon the causes which have been just exposed: the removal of such causes, howsoever induced, would give occasion to solid evacuations. But, whether the disease originates in the retention of fæces, or in increased action of the vessels of the inner membrane, the return of more consistent dejections is always a favourable indication.

"The great difficulty in the treatment of watery gripes, is to properly discriminate the cases in which purgatives are to be advised, from those in which they should be carefully abstained from. In general, where the child is suffering from copious watery stools, without there being much pyrexia, or pain of the abdomen on pressure, present, a dose of some brisk cathartic,—as calomel joined with rhubarb, scammony, or jalap, may be usefully administered; but, should symptoms of abdominal inflammation be concomitant, the more gentle laxatives, as cold-drawn castor-oil, rhubarb and magnesia, or syrup of senna, should be preferred, merely for the purpose of emptying the upper portions of the intestinal tube of any irritating matter which may be present. To fulfil a similar indication as regards the super-diaphragmatic portion of the digestive canal, an ipecacuanha emetic may be advisable. If, under this treatment, the stools become improved in appearance, and less frequent, the laxatives may be gradually decreased until they are finally abandoned altogether.

"Should the symptoms not yield to the aperients, the frequency of the stools being much increased without their character being altered, whilst the powers are rapidly sinking, the warm bath, fomentations, and anodyne friction, may be occasionally used, and an opiate clyster be given repeatedly during the day, in regulated doses; whilst the pulvis cretæ compositus, or any testaceous preparation, may be administered internally.

"By these means, even although the stools are unnatural, the inordinate secretion may be arrested; and gentle laxatives may subsequently carry off the offending matters." (P. 140—145.)

There remain five chapters: they treat of *Vomiting* and *Cholera*, of *Aphthæ*, of *Inflammation of the Stomach and Intestines*, and of *Intussusception*. We have read them.

In reviewing the general contents of the volume before us, we think two circumstances are evident:—1st, that the author has read many books,—that he has quoted frequently, freely, and fairly,—for he has always confessed his authorities; 2d, that on these quotations depends whatever value his work may possess, because, from his own personal observation, he has little or nothing to impart on the subjects of which he treats. Guided, therefore, by our invariable rule of giving, according to the best of our judgment, a fair account of the works which come before us, we would suggest that the present title-page should be cancelled, and the volume be called "Extracts from various Authors on the Diseases of the Stomach and Bowels of Children, collected and arranged by R. Dunglison, &c. &c."

---

ART. II.—*Transactions of the Association of Fellows and Licentiates of the King and Queen's College of Physicians in Ireland.* Vol. IV. —8vo. pp. 452. Dublin, 1824.

WE have already given, under the head of COLLECTANEA, several of the most interesting cases contained in the volume before us; and it is now our intention to present our readers with an analysis of those papers not hitherto noticed, and which, from their length, are not adapted to that department of our Journal. This volume, we are happy to say, is at least equal, in point of interest, to those which have preceded it, and affords a very satisfactory proof of the zeal and industry with which the different branches of the profession are cultivated in the sister kingdom; for, though professing to be the Transactions of the Fellows and Licentiates of the College of Physicians, the work includes, in fact, many observations and papers on subjects purely surgical, and is a good evidence of the kindly feeling which pervades the different branches of the profession in that country.

Without further preface, we proceed to the first article, which is a *case of Pregnancy occurring during the existence of a Tumor in the Womb,* and which tumor was removed after abortion had taken place. The facts of this case were shortly these:—A lady, twenty-five years of age, who lay-in of her first child in April, 1819, and was, for upwards of ten months afterwards, employed in an anxious attendance upon her husband during a protracted illness at Paris, returned with him to Ireland in August, 1820; and, on the 2d of September, consulted the author of the paper (Dr. BEATTY), in consequence of a swelling at the lower part of the abdomen, accompanied with a considerable pain on pressure. This swelling came on in May, but constantly disappeared during the menstrual periods, which were regular. On the 23d of the same month, the tumor was very evident, situated above the pubis; and on the 28th, Dr. Clarke was called in.

The os tincæ was found dilated to the size of a dollar, and in its opening was a large dense substance, of a regular smooth surface, connected closely with the internal surface of the uterus. The tumor extended up to near the umbilicus, and was so irregular, externally, as to have the appearance of two unequal tumors. It was judged right to attempt gradually to detach the tumor, and, for several days, regularly, Dr. B. introduced his finger, and endeavoured gently to separate the connexion between it and the uterus. In a few days, he succeeded to the extent of the full length of a finger, without giving pain, except at the posterior part, where he was stopped by what appeared to be a ligamentous attachment; the other parts giving way without pain or hemorrhage.

On the 11th of October, Dr. Clarke again saw the patient; but there was no perceptible difference, excepting the great increase of the tumor, which had reached far above the umbilicus, and was much more prominent in front.

Things remained in this state until the 11th of November, when the patient, whilst riding in her carriage, was seized with a sudden discharge of blood from the vagina. Dr. Beatty saw her in the evening, and found the parts within exactly in the same situation as in the previous month. There was some pain. She had lost her appetite, was thirsty, and felt generally unwell. At two o'clock the next morning, she miscarried: the embryo was entire, the membranes not being ruptured, and the placenta attached to them. The fœtus was not three months old; so that conception must have taken place about the middle of August, three months after she perceived what she called the lump in the womb.

Mr. Crampton was now consulted as to the removal of the tumor, but he dissuaded them from any active interference; and it appears that this mass was expelled, slowly and regularly, by the mere efforts of the uterus, so that, on the 17th, it was found at the os externum, where it remained partially attached, and was finally removed at twelve o'clock, in the presence of Drs. Evory and Marsh, and Mr. Crampton. No accident followed; the lady soon recovered, and in February, 1822, produced a healthy child. The tumor, which weighed nearly four pounds, had very much the appearance and texture of the placenta, but was more dense, and very vascular; the external surface was covered with a smooth membrane, and had the appearance of a highly vascular, gravid uterus.

The second paper we shall pass over: it simply records the *expulsion of a Biliary Calculus*, in a patient suffering under great derangement of the biliary organs, and which eventually caused death. There is nothing remarkable in the history of this case, excepting that the presence of this calculus was not so forcibly denoted by the symptoms as is sometimes the case: its size was, however, considerable, being one inch and a quarter in its larger diameter, and three-quarters of an inch in its shorter. Externally it was smooth, and of an oval shape.

The next communication which arrests our attention, is from the pen of "an experienced Physician," and relates to a species of *premature labour*, to which women are occasionally liable. We shall here confine ourselves to the facts of these cases, as related and confirmed by Dr. Beatty, leaving out entirely the speculative opinions of the writer, as to the acrimony of the blood, the existence of a venereal taint on the side either of the father or mother, although there are no symptoms of that disease to be detected, &c. &c.; speculations which, we are quite sure, can lead to no practical conclusion; and, therefore, we proceed to state the case, which is this:

"A lady, apparently healthy, conceives and carries her child in the usual way, till, about the seventh or eighth month of pregnancy, she by degrees ceases to perceive the motions of her child; and, in about ten days or a fortnight after this event, she falls in labour, and a fœtus, evidently dead for some time, is expelled. This often happens three, four, five, or six times in succession, or perhaps more frequently, to the same patient, and about the same period of pregnancy. The first time such accident happens, there has generally been some cause to weaken the patient, during gestation; but, in the subsequent instances, it rarely happens that any adequate cause can be assigned. Women, who have borne many healthy children, have sometimes fallen into this pernicious habit, and continued it for a length of time, and afterwards had living children. A memorable instance of this kind occurred in the lady of a viceroy in Ireland, about thirty years ago. In such cases, it is evident that miscarriage happens in consequence of the fœtus dying in utero." (P. 28.)

The solution of this difficulty is given by Dr. Beatty, who says, that, as early as the year 1789, his attention was directed to the subject, in consequence of a case he met with when resident assistant at the Dublin Lying-in Hospital; and he relates several cases in which women had been delivered of putrid children at the end of the eighth month; and, in all those cases, he was led to suspect a venereal taint to be the cause. His views were confirmed by the result of a mercurial treatment, to which these patients were subjected; and he adduces five strongly-marked cases, as confirming his view of the subject.

We must be excused from entering at greater length into the discussion which this communication is calculated to produce. We scarcely know what to say to Dr. Beatty's explanation of this affection, since, if we are to believe that a husband's having had the venereal disease some time before his marriage, although he is apparently well at the time, is sufficient to produce this accident to his offspring, we scarcely know who would be safe.

Still more extraordinary is the case of the lady of the cavalry officer, who was delivered of a putrid child in the eighth month of her pregnancy. In this instance the husband had contracted, whilst on the continent, a slight venereal complaint, of which the surgeon considered him cured before his wife joined him in France; and the author admits, that he could not detect any venereal symptom in the parents.

All we can say is, that Dr. Beatty's practice appears to have been eminently successful, though we do not quite understand his theoretical views; a matter, we are willing to confess, comparatively of little consequence.

At page 91, we find the history of *a case of fatal Result from Mercurial Ointment*, related by Dr. Crampton, accompanied with remarks upon that case by the same gentleman. This paper is worthy of notice, in several points of view. An attentive perusal does not, in our minds, fairly connect the death of this man with the employment of only three drachms of mercurial ointment; nor is the dissection such as to clear up our doubts. We also lament that the previous history of the case is not detailed at sufficient length to enable us to form an accurate judgment of the whole case. The following extract is all the information given to us of the man's previous state of health: the time he had laboured under these symptoms is not even hinted at.

"Joseph Jones, ætatis twenty, an engraver, naturally of a robust habit, was admitted into the Whitworth Chronic Hospital, on the 7th of November, 1821, with symptoms of ascites and general anasarca,—the eye-lids even being distended with fluid. Cathartic and diuretic medicines were exhibited under the direction of my esteemed colleague, Dr. Bryan, in one of whose wards he was placed. The expected relief not being obtained, on the 30th of November he was ordered the ung. hydr. ʒss. bis die, to be rubbed on the abdomen. After having used three drachms, his mouth became sore: the ointment was, of course, discontinued."

On the 8th of December, the parotid and submaxillary glands were much swollen. Leeches were applied; and, on the 10th, blood was taken from the arm. On the 12th, fifteen more leeches were applied to the tumid parts; but, on the 14th, as his breathing became oppressed to an alarming degree, he was removed to a large empty ward, which affording only temporary relief, he was put into a slipper-bath in the evening, and again bled to the amount of fifteen ounces. On the two following days, blood was discharged with the saliva. The bowels were kept open by a laxative draught; though he appeared better, on the same night the discharge from the mouth ceased, the swelling of the face subsided, and he died with every appearance of apoplexy.

Dissection took place ten hours after death; but there was no effusion in the brain. The liver was diseased; the gall-bladder half full of green bile; the mucous membrane of the small intestines, particularly of the ileum, was of a bluish green colour, ulcerated in many parts, or rather appearing as if corroded by some active chemical substance. This condition was not observed in the large intestines, but green-coloured fæces adhered to the inner surface of the intestinal canal.

Now, we cannot here be satisfied that the appearances found are at all, or in any great degree, attributable to the three drachms of mercurial ointment; and we are quite sure that the treatment adopted, under the impression that mercury was acting deleteriously upon the system, was calculated rather to render it more active than to allay it. The bleeding, warm baths, &c. were, we conceive, not indicated; and the more especially as the mercury had produced its ordinary and legitimate effects upon the salivary glands; in which cases, we have seldom found the dangerous results, as described by Mr. PEARSON and others, to take place: sudden death by what has been called mercurial erethism generally occurring where that medicine has been long accumulating in the habit, without any of the more usually marked symptoms of its action.

Our author's reasoning upon the appearance of the intestines, appear to us to be overstrained. Such ulcerations in a diseased condition of the liver, which may have existed, for aught we know to the contrary, for an indefinite period; the quantity of green bile in the gall-bladder, connected with the green-coloured fæces in the intestinal canal, are all explainable, we conceive, without any reference to the small quantity of mercury employed. Our limits will not permit us to pursue this subject further; but we confess that we perceive no analogy between the case recorded above, and that to which the author alludes in the subsequent portion of his paper; and we are always inclined to discourage general conclusions deduced from a solitary fact.

Passing over a case of *misplaced Viscera*, which affords no particular point of interest, we find, at page 131, the detail of *three cases of Irritable Bladder*, cured by an infusion of the buchu leaves. The disease which Dr. M'DOWELL describes, is one by no means unfrequent, and very distressing. The second case affords an excellent specimen of the symptoms usually accompanying it, and, being compressed into a short compass, we feel disposed to transcribe it.

"Philip Dwyer, aged sixty-seven years, sallow complexion, emaciated; ill for three years. Complains of severe pain in the pubic region, particularly before he passes water. Great irritability of bladder, passing water in small quantities every quarter or half hour during the night: during the day, can occasionally retain it for two hours. Less irritability when using much walking exercise: when sitting, is affected with a stinging or scalding sensation in the prostatic region. Urine generally white or muddy. Frequently passes a large quantity of a slimy, pale yellow-coloured mucus, voided with great difficulty, and soon putrefying: is much relieved by its expulsion from the bladder. Is greatly debilitated, and has lost much weight. Tongue loaded with yellowish mucus; thirst; no appetite; bowels generally constipated, stools black. No enlargement of the prostate could be felt.

"*Previous history.*—Never had gonorrhœa; has been a temperate liver. The disease commenced three years ago, first with slowness and difficulty in passing water, which was followed by frequent micturition. He attended the Talbot Dispensary for five months, and left town apparently cured. He relapsed, however, in a month, and returned to the Dispensary, May 13th, 1822. He was ordered a pint of the aq. calcis daily, twenty drops of the muriated tincture of iron three times daily, an opium suppository (three grains) every night, and purgative pills to be taken occasionally." (P. 135, 136.)

This man commenced taking the infusion of buchu leaves on the 29th May, and was well by the end of July. It is scarcely necessary to say, that, when these symptoms of irritable bladder are dependent upon diseased prostate or the presence of a calculus, the above means cannot be supposed to have any influence over them.

If we omit to notice Dr. Evanson's cases of *Hydrocephalic Fever*, which occur next in order, it is not that we consider them as deficient in interest; but the very nature of our analysis obliges us to pause principally where any thing novel, either in theory or practice, strikes us as commanding attention, or deserving consideration. And, for the same reason, we shall not extract any portion of Dr. Teeling's case of *Suppression of Urine*, which, like all the cases of the kind which have occurred to us, either in books or in practice, was followed by death.

A letter on *Variola after Vaccination* next presents itself to our notice, from Dr. Clarke to Dr. Brooke, in which the Doctor seems to wish it to be understood, that the numerous cases of eruptive disease after vaccination are not cases of variola; and he refers us to Dr. Cullen's definition of the latter disease, the distinctive character of which is, that "*spatio octo*

diarum in *suppurationem* abeunt;" and he appears to think that nothing should be admitted as small-pox that will not stand the test of this definition. For our own parts, we think it much better to admit at once fairly that variola after vaccination is a common occurrence; but that the absence of secondary fever, &c. have so far altered and modified its features, as to disarm it of its malignancy: and, really, to add an argument in addition in favour of vaccination, we are extremely gratified to perceive, by the return annexed to this letter, that the number of persons vaccinated in 1822 greatly exceeded that of the previous year, and that upwards of 3200 packets of lymph had been distributed to practitioners in general during that year.

Dr. Pickel's case of *Insects in the Stomach*, and Mr. Adam's cases of *Tumors in the Neck*, have been detailed so much at length, that we do not deem it necessary to dwell upon them, as they are probably familiar to most of our readers.

Dr. Francis Barker has favoured us with an account of his experience of the *Sulphate of Quinine*, as a remedy in *Intermittent Fever*; and, as this medicine is deservedly rising in public estimation, we cannot do better than give the author's account of its effects in thirty cases, in which he employed it.

"On inspection of the preceding table, it becomes evident that the sulphate of quina is an effectual remedy for intermittent fever, and succeeds in cases which resist the bark. Of thirty persons treated with this remedy, not one has resisted its use, and in a majority of the cases the disease ceased within a day or two after the first dose was taken. Very small doses were employed, as a part of the object was to determine the smallest quantity capable of effecting a cure; and, although this has not been ascertained with precision, yet it was proved that a grain, or less, taken three or four times a-day, was as efficacious as larger doses. In one of Dr. Morgan's cases, half a grain three times a-day suspended the paroxysm for eight days. In no case did it disagree with the stomach, an effect often attendant on the use of Peruvian bark, especially when taken in large doses; and a quantity of the sulphate of quina, much exceeding that requisite for the cure of the disease, may be taken without inconvenience.

"If we reject those instances given in the preceding table, in which a very large quantity was employed, we shall find the average quantity of sulphate of quina, requisite for the cure of intermittent fever, to amount to somewhat more than nine grains.

"The advantages arising from the introduction of this remedy into medical practice, are these: it is efficacious, as appears from its power of curing intermittent fever, and in this respect it is, at least, equal to Peruvian bark; it contains, in the dose of a grain or a little more, a quantity of the essential curative ingredient of the Peruvian bark, equivalent to a full dose of that substance; it does not overload the stomach, or disagree; and in a small bulk may be contained a quantity sufficient for the supply of even a large army, in situations productive of intermittent fever." (P. 270, 271.)

Six cases of *Tetanus* are related by Mr. CARMICHAEL; one of which terminated successfully. The author of this communication does not pretend to give any new view of this intractable disease; but still the cases are valuable on several accounts: in the first place, the dissections of those who died appears to have been carefully performed; and, as far as two cases can be said to go, (for, in the other three fatal cases, the friends would not permit any examination of the bodies,) they prove that the spinal canal is by no means generally implicated in the disease. They also demonstrate the fallacy of all the remedies hitherto employed for the cure of this affection; while they afford a remote hope, that the vigorous and extensive application of the tartar-emetic ointment may, under certain circumstances, be attended with happy results; since it appears that the patient who recovered had a very extensive crop of pustules produced upon the back, from the employment of that remedy: though it is right to say, that Mr. C. attributes this man's recovery, in some measure, to the spirits which he took at his own request.

In the last case recorded, the tartar-emetic ointment was attempted to be used; but the recurrence of the spasms afforded such an obstacle to its application to the back, that its failure in this instance ought not to discourage us from repeating the experiment upon as large a surface of the body as possible.

The remedies tried in the unsuccessful cases were bleeding, opium, baths, tobacco in the form of a vinous tincture, purging, and mercurial inunction; all which powerful agents appear to have been employed in the most energetic manner.

We regret that we cannot afford room for the insertion of Mr. Carmichael's general observations upon this complaint, and with which the paper concludes. We observe, however, that the author is of opinion, that the sympathetic nerve is more immediately the seat of the disease; those muscles being chiefly affected whose nerves communicate extensively with the sympathetic: the muscles of the extremities, which derive their nerves from the spinal marrow, being but little affected, and the senses and intellectual functions remaining unimpaired. With regard to remedies, our author thinks that opium, though it cannot cure, greatly alleviates the distressing symptoms: of tobacco, he has nothing favourable to report; and respecting the tartar-emetic ointment, he justly says that, if it is to do good, it should be applied upon the abdomen rather than the back, as there it is difficult to introduce it, in consequence of the spasms. He never saw either the cold or warm bath of the least use; and the same opinion is given as to blood-letting. Mercury, Mr. C. says, has never been found useful in tetanus itself, but eminently so in those spasmodic twitchings of the muscles of the limbs in patients of irritable habits, who are suffering under wounds of those parts. Finally, our author is inclined to think that something may be expected from the employment of stimulants. In the successful case which he has recorded, spirituous liquors were freely drank; and he is inclined to ascribe the recovery in some degree to them, as well as to the tartar-emetic ointment.

It appears, from a note annexed to the paper, that he was not aware, when he suggested the use of sulphuric ether in large doses, that Dr. Reid had before observed that a dose of ether caused a relaxation of the spasms in a case of tetanus.

In any future case of tetanus, therefore, Mr. Carmichael says, he shall feel no hesitation in using alcohol, in any of its various combinations; in exciting extensive counter-irritation by means of the tartar-emetic ointment; using ether and opium freely by the mouth, as well as by enema; and purging with castor-oil and oil of turpentine.

Mr. Ryall's paper on the *Purulent Ophthalmia of new-born Infants*, is deserving of commendation, both for the accurate description he has given of the disease, as well as on account of the soundness of his practical directions as to the cure. This, if properly managed, we have generally found no very difficult task; and we cannot speak too highly of the stimulating plan of treatment, as recommended by this gentleman. We are not quite so well satisfied with his explanation of the origin of the disease; and cannot help believing and hoping that the application of morbific matter from the mother to the child has less to do with it than he appears to think. Generally speaking, the purulent ophthalmia of infants terminates favourably, though slowly; whereas, in all those cases of gonorrhœal ophthalmia which we have witnessed, the disease has run its course with the most fatal rapidity, and nothing short of the most vigorous plan of treatment can arrest its progress.

Two points of Mr. Ryall's practice demand attention: the application of all washes, of the stimulant kind especially, by means of the syringe inserted between the lids; and the great superiority of the lotion composed of lunar caustic, in the proportion of two or three grains to the ounce of distilled water, over every other form of wash.

A valuable practical caution concludes this paper. We are directed to ascertain, as early as possible, the condition of the ball of the eye; but, in doing this, to be careful not to employ more force than necessary, and not to use any speculum for this purpose, but gently to raise the upper eye-lid with the thumb of one hand, depressing the lower one with the index-finger of the other.

Two cases of *Diabetes* are shortly recorded by Dr. SHARKEY, in which that disease was successfully combated by the phosphate of soda, taken in doses of a drachm three times in the day; the patients not having been restricted,—or rather, indeed, living exclusively upon vegetable diet, during the whole period of their cure. This is in direct contradiction of the plan recommended by Dr. ROLLO.

Dr. O'BIERNE is the author of the next communication, which is highly important, should subsequent observations confirm the author's evidence. The paper is on the *use and advantages of Tobacco in Dysentery.* Dr. O'Beirne is already known to the profession, as advocating the use of tobacco in cases of tetanus and epilepsy; and he informs us that, whilst prosecuting these inquiries, he was struck with the energetic action caused by this plant on the circulating fluids, producing the immediate effects of copious blood-letting; which effects disappeared in a day or two after discontinuing its use, thus leaving the patient nearly as strong as before. On various other occasions, observing its power of relieving pain and removing constipation, he conceived that important advantages might be obtained from its employment in dysentery.

It is right to mention, that the cases detailed appear to us genuine specimens of that disease, of which the author had ample experience during the Peninsular war; and that six out of seven of the cases are certainly almost, if not wholly, indebted to the tobacco for the cures performed. The seventh case does not seem to us to be so unequivocal a specimen, since other powerful remedies were employed; and we are by no means convinced that the benefit derived was fairly attributable to the tobacco. That we may put our readers in possession of the mode of employing this remedy, we publish, without abridgment, the sixth case, which is the most severe of those recorded:—

"Mary Neill, aged forty-five, half-starved, and nearly naked, admitted into the Charitable Infirmary, Jervis-street, on the evening of the 31st of December, 1823. Ten days previously, having slept in a damp cellar, she was seized with rigors, nausea, vomiting, tormina, tenesmus; great pain of abdomen on pressure; frequent discharges of blood, jelly, and wind, as she described. On admission, her countenance was pale; skin dry and warm; tongue white, furred, and rather dry; pulse 98, small, and rather hard; urine scanty and high coloured; abdomen tense, and painful on pressure; nausea, and occasional vomiting; had been at stool more than twenty times during this day. Discharges, being examined, consist of blood and mucus: they were attended with the expulsion of much flatus.

An ounce of oleum ricini ordered to be given; and to foment the abdomen with an infusion containing three ounces of tobacco in two quarts of boiling water, and allowed to stand for twenty minutes before use.

January 1st, 1824.—The first application of the stupes, to use her own language, made her very weak, turned her stomach, and gave her a belly-ache; pulse now nearly as before; tenesmus almost as urgent; abdomen less painful on pressure. The oleum ricini ordered to be repeated, and the tobacco fomentation to be afterwards used, until considerable weakness of the head and stomach should be produced.

2d, nine o'clock A.M.—Passed some hard fæces last night; tenesmus not so urgent; less blood in the stools; vomiting less frequent; feels very weak; pulse not so hard, and 96. The oleum ricini and tobacco stupe to be repeated.

Seven o'clock P.M.—Has had, since morning, a free, feculent stool, slightly tinged with blood. The countenance now less pale and anxious; pain on pressure of abdomen greatly relieved; has had no vomiting since the feculent stool; also slept two or three hours since, and had some wish for food. Pulse 86, soft and full; tongue cleaning. Five grains of calomel, followed by half an ounce of the sulphas magnesiæ, in two ounces of water, were ordered to be given; and, in half an hour after, to resume the same fomentation.

"3d.—Has had, without griping, frequent and copious discharges from the bowels, of a still more natural appearance, with less blood, and no mucus. Passed urine in large quantity, and slept well during the night. Pulse now 86, full and soft; no pain on pressing the abdomen. Ordered merely to use the tobacco stupe during the day and night.

"4th.—Stools less frequent, and partly feculent and mucous, but without blood; abdomen soft, and free from pain on pressure; pulse as before; tongue still furred and dry. The oleum ricini and the same fomentation to be used.

"5th.—Had four motions since yesterday, each of hard fæces and mucus, but no appearance of blood; had slight tormina last night, which was removed by the tobacco stupe, and for which she called herself. Appearance of the tongue and countenance now much improved; skin soft and moist; pulse as yesterday; no tormina, tenesmus, or pain on pressing the abdomen. The same treatment to be repeated.

"6th.—Had three feculent and mucous motions since yesterday,—one slightly tinged with blood: these sort of discharges continue today, and are accompanied by much flatus. The calomel and sulphas magnesiæ, as once before, and the tobacco stupe, directed to be used.

"7th.—Much improved: stools frequent, feculent, and mixed with very little mucus. Treatment as yesterday.

"8th.—Improving rapidly; scarcely any mucus in the stools.

"9th.—Neither blood nor mucus appeared from this period; the motions became gradually fewer, and more natural.

"Until the 9th, the diet was similar to that in the preceding cases, with the exception that she was allowed some warm flummery and cold milk from the 5th. On the 9th, she had beef-tea and bread; from the 10th, light animal food.

"She was discharged cured on the 12th, and with more strength than might, under the circumstances, be expected."

We shall offer no apology for this long extract. The subject is extremely interesting even in a political point of view, since, as the author justly remarks, armies and navies have been rendered useless by attacks of this disease; and, under the most successful mode of treatment hitherto adopted, the patient is extremely reduced, his convalescence tedious, and he is not restored to the service frequently for months. The remedy is also every where procurable; another valuable consideration, to the army-surgeon especially.

A *Report of the Fever lately prevalent in Galway*, by Dr. Robert Graves, concludes the volume. This report is more statistical than medical, though it confirms, in every leading particular, the observations of those who have witnessed the invasion of fever among armies,—particularly the instances of Sir J. Moore's campaign, and after the retreat from Burgos. In every affliction of this kind, want of food and clothing, great bodily fatigue, and the depressing passions, have been found sufficient to excite fever, which, when once prevailing, is aggravated in its form, and rendered, of course, more frequently fatal by the crowded state of hospitals, by the great accumulation of sick upon one spot, and by the very same moral causes that, in the first instance, contributed to bring it into existence. We were particularly struck, in reading Dr. Graves' able report, not only with the coincidence of causes, but likewise the great similarity of symptoms. Diarrhœa, or rather dysentery, we always observed, in the Peninsular war, to be the precursor of fever; and we always found affections of the bowels a serious, generally a fatal, accompaniment of this form of fever.

It is in vain to look for the causes of this disease in imported contagion, or atmospheric influence: the history of the world affords but too many examples where great public calamities, by inducing famine, have laid the foundation for pestilence. Gibbon, though no medical man, has made the same remark; and we cannot better express ourselves than in the words of Dr. Crampton, that, "whenever it shall please Providence to afflict a populous country with a scarcity of provisions,—while there exist also additional causes to depress the mind, and occasion despondency,—and, moreover, when cold, wet, and ungenial seasons are superadded to other causes of distress,—an unusual number of fever-cases will be the result in those places where the population is most dense, and where the habits of the people are most negligent."

### DIVISION II.

### FOREIGN.

ART. III.—*Memoire sur la Nevrite Puerperale, ou Inflammation des Nerfs chez les Femmes en Couche; d'apres des Observations de la Maternitè.* Par M. ANT. DUGÈS.

*Memoir on Puerperal Neuritis, or Inflammation of the Nerves in Child-bed; from Observations made at the Maternitè.* By M. ANT. DUGÈS.

THE Memoir which we have selected for the present Article is one of considerable interest, and, as we have little of our own to add to the remarks contained in our review of the works of Mr. HUTCHINSON and others, (Sept. and Oct. 1822,) we shall content ourselves, at present, with an analysis of M. Dugès's paper, and the few observations which its perusal has suggested.

Inflammation of the nerves, according to our author, is an affection as yet but little known,—at least in its acute form. The dissertation of COTUNNIUS treats only of chronic inflammation, (ischias nervosa) which he, probably, was the first to distinguish from gout and rheumatism, with which this disease was generally confounded, under the name of sciatica. Most modern writers, although they admit the distinction, seem to refuse to this form of sciatica (nervosa) the character of inflammation, and range it with tic douloureux and similar affections. This doctrine is regarded by the author as generally correct, but somewhat too exclusive; and he agrees with Cotunnius in referring to inflammatory action, more or less chronic, the infiltration described by the latter. In the acute form, the inflammatory character, as might be expected, is more decided, and presents more unequivocal traces on anatomical examination; but the cases of this complaint are so rare, and these so rarely fatal, that it is not surprising, that our information on this point should be vague and unsatisfactory.

REIL and PORTAL assert, that they have found marks of inflammation in the cerebral nerves, BRESCHET and LOBSTEIN (see our review of the work of the latter, De Nervi Sympathetici Fabrica, &c. Feb. 1824,) in the pneumo-gastric and splanchnic, and further observations of a similar kind have recently been made by M. Martinet.* M. Dugès thinks it probable that the names of rheumatism and gout have been given to neuritis, whether acute or chronic; and acknowledges himself to have been guilty of a similar error on two occasions. He gives the cases to which he alludes at some length, but the one patient died without being examined after death, and the other recovered; so that in neither instance was the truth of our author's conjectures verified by demonstration. We, therefore, hold ourselves absolved from quoting them. When neuritis occurs in child-bed it is regarded, says our author, as neuralgia, depending on compression of the nerves during labour,—then, if the inflammation extends to the neighbouring parts, the abscess thus formed absorbs all our attention, while the state of the nerve is neglected. From this remark it is obvious, that M. Dugès regards puerperal neuritis as much more frequent than it is generally supposed to be; he informs us, however, that this idea is neither new to him nor rashly brought forward, and with a view of developing more fully his ideas upon the subject, we shall follow him through his somewhat too numerous subdivisions. They are as follow:—1st, Simple, or circumscribed neuritis. 2d, The œdematous, that is to say, one which causes an effusion of serous fluid not only into the tissue of the nerve, but likewise into the surrounding cellular membrane. 3d, The phlegmonous, or that which brings on phlogosis, and frequently suppuration of the nerve and neighbouring parts. 4th, The œdemato-phlegmonous, which participates in the character of the two preceding; and 5th, The gangrenous, which brings on mortification of the parts surrounding the inflamed nerve. We shall analyse the account given by our author of each of these successively.

*Circumscribed Neuritis.* — This variety, according to M. Dugès, has almost always been attributed to compression of the nerves. But this is not universally the true explanation. It has very frequently been confounded with neuralgia, because no swelling was perceptible; but the observations of M. Martinet prove that inflammation of a nerve may exist without swelling;—in addition to which, our author refers to the diagnostic character above mentioned.

"Characters (he observes) in general so obvious, that the inflammation cannot be denied, even although we should not be able to find, on examination after death, that redness and infiltration which are constant when the disease has been of some duration, as I have more than once established under the inspection of M. Chaussier, who, I believe, admits the inflammatory character of these painful affections. I have likewise sometimes seen the nerves in appearance healthy, when peri-

* *Revue Médicale,* June 1824.

tonititis has cut off, in the course of a few days, a woman affected with circumscribed neuritis."

The author states that this variety, the duration of which sometimes scarcely exceeds five or six days, and which is often limited to some transient stitches, causes much more acute lancinating pains than those which occur in the *phlegmonous* neuritis:—It likewise yields much less readily to the abstraction of blood; opiates seldom relieve it; but baths generally avail, even in the most obstinate cases. The sciatic nerve is the most common seat of this affection, which sometimes limits its effects to the region of the pelvis, and at others extends to the thigh, leg, and even to the foot.

*Œdematous Neuritis.*—This variety is said to be less frequent than the preceding, and also less painful, quickly giving place to œdema of variable extent, sometimes occupying all the lower extremity,—always active in the first instance, and afterwards assuming a passive character. The pain quickly subsides, but the œdema remains obstinate, and then some tumefaction is almost always to be found in the lymphatic glands of the groin, if the neuritis has occupied the crural nerve. This variety is easily confounded with active anasarca: but the anasarca in this is only secondary, and depends on the increased effusion caused by the irritation of the nerve, and consequently of the parts on which it is distributed. The serosity is in general a little turbid, and the nerve sometimes, in addition to this infiltration, shews some spots of purulent matter.

*Phlegmonous Neuritis.*—This is not confined to the inferior extremities, but occurs in the arm or forearm of women in child-bed: more frequently, however, it affects the crural and other nerves in the same neighbourhood. It may readily be mistaken for simple phlegmon, and the abscess which results, marks the state of the nerve so much, that even in the dead body it alone fixes the attention of an observer not aware of the character of the disease,—an error the more likely to occur, as the woman in child-bed, like others, are liable to true phlegmonous affections. The following are the distinguishing characteristics given by M. Dugès.

"1. The pain follows the course of the nerve, and is neither deeper situated nor more superficial:—2. This pain is more extended in length *(en longueur)*, more acute, of a more insupportable character, of longer continuance, than in an ordinary abscess:—3. The swelling is likewise more extended lengthways, being always directed longitudinally:—4. The swelling always precedes the redness of the skin, and, on the contrary, follows the pain; it presents more inequalities, and is harder than in a simple phlegmon:—5. A rigor pretty constantly precedes the commencement of both, but that which ushers in the neuritis is longer, more intense, and more fatiguing; the fever which follows is also more violent."

The author states, that, on examining the body, he has found an infiltration of concrete pus into the diseased nerve, occasionally into the surrounding cellular membrane, and more rarely into the muscular tissues. In other cases he has seen more considerable collections of matter,—the pus resembling that found in ordinary abscess.

*Œdemato-Phlegmonous Neuritis.*—This appears to be nothing more than the phlegmasia dolens of other writers, although our author is certainly not correct in his assertion, that almost all the moderns agree with Dr. ALARD in regarding it as inflammation of the lymphatics. Be this as it may, M. Dugès has adopted the opinion, (which however is not new,) that it consists essentially in an affection of the nerve; the reasons for this theory being, that the pain commences in the pelvis, and is accompanied by a sort of numbness in the thigh; that this pain precedes by several days the swelling and enlargement of the lymphatic glands, which takes place at a much later period, and often resembles a tense cord, sometimes having knots upon it; that the swelling, as well as the pain, always proceeds from above downwards, while other œdematous effects have an opposite course, beginning at the extremities and extending towards the trunk; that tumefaction does not always take place; whereas their enlargement and distention are the characteristics of diseased lymphatics; that the pain is more acute than in any lymphatic disease; that the symptoms run their progress with greater rapidity; and, lastly, that after the recovery, a sort of palsy frequently continues for a considerable time. He is further of opinion, that, generally speaking, we ought to suspect the lesion of a nerve, whenever there exists acute pain in an organ which is naturally but little sensible. Our author, in confirmation of his opinion, quotes some remarks of M. BOER's, and we are willing that he should have all the benefit of this authority.

"Sometimes (we give the words of M. Boër,) at the moment when we least expect it, women in child-bed are seized with a violent pain in the groin or iliac fossa, but still more in the anterior part of the leg, and often over the whole extent of the lower limb. It occurs indifferently in those who have had a very favourable as well as those who have had a severe labour. The pain commences towards the upper part of the limb, and extends towards the lower. During the first days there is neither tumor, nor redness, nor induration, but the parts cannot be moved without extreme suffering;—occasionally no intumescence supervenes at any period. *The cause of this affection appears to be, pressure exerted by the head of the fœtus on the nerves of the pelvis.*"

This is all that M. Boër says about the matter, and the only part of the quotation in any degree applicable to the doctrine of M. Dugès is that which we have placed in italics. Even this we suspect will appear of little value when we consider that pressure upon a nerve is by no means necessarily accompanied, or even succeeded, by its inflammation. Indeed, it is but justice to M. Dugès to state, that he seems little satisfied with the evidence he has brought forward to prove that phlegmasia dolens consists essentially in inflammation of the nerves, but comforts himself by declaring, that at all events there is inflammation *somewhere*, and with this unlimited qualification few will dissent from his opinions. ("Du reste, quelqu'en soit le siege il est certain du moins que cette maladie est inflammatiore.")

*Gangrenous Neuritis.*—On this branch of the subject our author gives no satisfactory information. Indeed he admits that no case deserving this name is likely to occur if the term be limited to mortification of the nerve itself, without that of the neighbouring parts;—but he thinks otherwise if we extend it to those instances of neuritis which give rise to sphacelus, in which he backs himself with the testimony of TOMMASINI, who holds all phlegmonous inflammations terminating in gangrene, to depend on the nerves participating in the disease. Our author likewise conjectures, that the sphacelus so frequently observed in epidemics, produced by the ergot, may depend upon the same cause.—But these, after all, are mere conjectures.

The author follows up his general description with the detail of particular instances, and, with a view of further illustrating his doctrines, we shall give an example of each of the varieties of the disease above mentioned.

*Case of circumscribed Crural Neuritis.*—Mary M. Gamet, aged thirty-five years, and of lymphatic temperament, had been ricketty during her infancy. She was pregnant for the eighth time, and the narrowness of the pelvis rendered the turning of the fœtus unavoidably necessary. This was accomplished on the morning of the 3d of January, 1812, after she had been twenty-four hours in labour. The operation was immediately followed by hemorrhage, and afterwards by spasmodic rigor; lastly, by fever and pains in the loins, the right iliac region, and the epigastrium. The following day the pain of the epigastrium subsided, while that of the lumbar and iliac regions increased, and extended to the anterior part of the right thigh. The fever was intense, but the lochial discharge abundant. The fifth day, notwithstanding the continuance of the pain, the secretion of milk took place, and the lochia continued to flow. Cataplasms, moistened *(arrosès)* with an aqueous solution of opium, did not produce any relief. Nevertheless the pain and fever gradually went off during the following week:—after a time the pain was confined to the iliac region, and at length, about the sixteenth day, it disappeared altogether. The twentieth day a violent rigor took place, and continued for several hours, being followed by a return of the fever and abdominal and iliac pains. These symptoms continued during twelve or fifteen days without any other treatment than diet and —— -expectation! (sans qu'on leur opposât d'autre traitment que la diète et l'expectation). They gradually went off, and this patient left the hospital in good health the thirty-eighth day after her confinement.

*Case of Œdematous Sciatic Neuritis.*—Charlotte Dotigè, aged twenty-three years, feeble and leuco-phlegmatic, was delivered of her first child, by a natural labour, the 5th of July, 1813. The child weighed seven pounds. During the first six days transient fever and diarrhœa. The third day, secretion of the milk:—the fifth, colic, relieved by a laxative:—the seventh, towards evening, slight rigor, restlessness, and diarrhœa:—on the morning of the eighth, pain in the course of the sciatic nerve of the left side, and, at the same time, infiltration of both lower extremities, particularly the left; the tenth day the pain encreased, notwithstanding the application of anodyne fomentations. After a few days the fever ceased, but the pain continued, as well as the swelling of the left limb; both these symptoms, however, gradually disappeared by the seventeenth day, although all treatment had been discontinued. M. Dugès is decidedly of opinion that the disease could not have been caused by any pressure upon the nerve during labour, as it did not manifest itself till seven days after the accouchement.

*Case of Crural and Cubital Phlegmonous Neuritis.*—Mad. Berton, aged thirty-nine, of sanguine temperament, was brought to bed, at the full period, of her fourth pregnancy, the 7th of May 1812, after a labour which lasted four hours. During the first night she had a rigor of a quarter of an hour's duration, loss of sleep, fever, and pain of the abdomen. A dose of ipecacuanha excited bilious vomiting, and various liquid stools, without any benefit. In the night of the third day, she had a similar rigor, followed by similar symptoms. The night following, another rigor took place, and peritonitis became distinctly marked, and accompanied with vomiting of black matter; the lochia continued. Next day, fever and delirium. On the eighth day, the abdomen was discovered to be more painful towards the groin than elsewhere;—in the right groin was a diffuse swelling of some extent. Next day the tumor was red, very painful, and of some extent: the patient complained of violent pain in all the limbs, and a considerable swelling made its appearance at the anterior and inferior part of the fore-arm in the course of the cubital nerve. The pulse became weaker, the delirium encreased, and, on the tenth day from the commencement the general pains, as well as those of the groin and fore-arm, continued.—The patient gradually sunk, and died in the night. The examination after death presented the effusion usual in peritonitis: in the groin and fore-arm pus was found infiltrated in the sub-cutaneous and inter-muscular tissues.

In this case we must be allowed to say, that there is no proof whatever of the pain in the groin and fore-arm having depended on any thing more than common phlegmonous inflammation,—the nerve is not even mentioned in the dissection, an omission which we think ourselves entitled to interpret, by supposing that there was nothing found to strengthen the doctrines of our author. Of the practice we would willingly say nothing, did we not conceive it to be our duty on every occasion to protest against that which is bad, and to point out the weakness of that which is nugatory and inert. But we anticipate:—our readers will expect, perhaps, that we have to object to the general bleeding, as having been insufficient, or carried to an unwarrantable extent,—to the number of leeches,—to the strength of the purgatives,—or to the doubtful administration of turpentine, as recommended by M. Martinet. It is to none of these, however, we object,—for none of these were employed. The whole treatment consisted in giving the patient whey, camphor, julep, tisan of mallows, and rubbing mercurial ointment on the abdomen; together with blisters to the legs the day before death, when the woman was rapidly sinking. In short, the patient was— not killed it is true, but allowed to die without any rational effort to save her. The French are good pathologists, but it is impossible to read their journals without being forcibly struck with the general inertness, and often with the futility and absolute childishness of their practice.

*Case of Œdemato-Phlegmonous Crural Neuritis.*—Marie Dumon, aged twenty-two; had been chlorotic during her pregnancy, and miscarried at the end of the sixth month, the 14th of Nov. 1811, after rather a severe fall. The labour lasted ten hours, and the child weighed three pounds. Next day she had fever without any rigor; slight pain in the abdomen; deep seated pain in the course of the crural nerve, followed by swelling and infiltration of the thigh, and then of the leg on the left side; the tumified parts gradually became red, and assumed an erysipelatous appearance. The lochia natural. The third day the same state continued, nevertheless the secretion of milk took place,—no fever. This kind of œdematous erysipelas diminished but very slowly, and did not entirely disappear till the end of a fortnight. Twelve years afterwards this woman, pregnant for the third time, returned to be confined in the hospital, Sept. 4, 1824. Two children, weighing together ten pounds and four ounces, were extracted by the feet :—on the fourth day secretion of milk, and, at the same time, acute pain in the right sciatic region ;—intense fever. The application of twelve leeches produced no change, the pain extended itself along the course of the nerve to all the back part of the thigh. Cupping, cataplasms, vapour baths, &c. procured only temporary relief. A scald made accidentally on the abdomen encreased the pain and fever, which assumed a typhoid character, and carried off the patient on the fourteenth day.—No detail is given of the appearances after death.

*Case of Gangrenous Sciatic Neuritis.*— —— Jaquet, a strong woman, aged forty-one, the mother of nine children, and again in the seventh month of pregnancy, was taken, the 16th of Dec. 1820, with rigors and febrile heat, accompanied with uterine hemorrhage, which rendered it necessary to have recourse to plugging. It was previously ascertained that the hemorrhage was caused by the placenta being situated over the mouth of the uterus. The labour had lasted three days when the fœtus was turned ;—the patient lost much blood, and remained in a state of intense fever and alarming weakness. Next day the fever increased, and intolerable pain came on in the left buttock, near the origin of the sciatic nerve. These pains were followed after a short time, by the development of a livid clammy (*nateuse*) tumour. Emollients and narcotics produced no abatement of the symptoms. The pulse retained its frequency while it became diminished in strength; the prostration and paleness were extreme, and, notwithstanding the use of bark and wine, the patient died on the 24th, being forty hours after her delivery. The body was examined thirty-six hours after death. The atmosphere was dry and cold, notwithstanding which, putrefaction had commenced, and the textures were infiltrated with putrid gas, which was likewise contained in the right cavities of the heart and in the uterus ;—the spleen was softened and converted into "putrilage." The blood contained in the heart formed colourless clots of a dirty appearance,—the serosity contained little particles like grease. In all the great veins, particularly in the inferior cava and its branches, nothing was to be found but a muddy (*boueuse*) fetid matter, of a deep brown, and resembling human excrement. In the smaller veins, the blood was red and fluid. The glutei muscles, and all the cellular tissues in the vicinity of the sciatic nerve, were converted into putrid matter similar to that contained in the great veins. At a greater distance from these parts, the cellular membrane was infiltrated with reddish serum, and a similar fluid was found in the tissue separating the nervous fibres of the sciatic trunk. The skin was purple and destitute of epidermis.

The conclusions at which M. Dugès arrives are as follow. "1. That inflammation of nerves is more frequent than the silence of authors on this subject would lead us to suppose. 2. That many of the maladies in childbed-women, designated as neuralgia, phlegmasia dolens, inflammation of the lymphatics, &c. appear to be the immediate result of inflammation of the nerves. 3. This opinion, however, requires further examination, and new investigations are necessary to establish, in a decided manner, the distinctive characters of neuralgia properly so called, of inflammation of the lymphatics, veins, &c. and of that of the nervous trunks of the limbs." Although several of the cases given by our author appear to us unsatisfactory, yet there seems some truth in the general distinction which he wishes to draw between *neuralgia* and *neuritis*, and we are not without hopes that this article may meet the eye of some of the gentlemen who have furnished the numerous and instructive cases of diseases of the nerves recently published in this Journal, and lead them to direct their attention to the subject.*

### MEDICAL AND PHYSICAL INTELLIGENCE.

* Since writing the above, we have received the Number of the *Revue Medicale* for September, in which a note is inserted by M. Dugès, intended to shew that he has not confounded inflammation of the nerves with inflammation of the veins. It is sufficient to remark, that he has urged nothing in this explanation which in any degree alters the opinions we have given above.

